# Longitudinal Estimation of the Clinically Significant Change in the Treatment of Major Depression Disorder

**DOI:** 10.3389/fpsyg.2018.01406

**Published:** 2018-08-06

**Authors:** Cristina Cañete-Massé, Maribel Peró-Cebollero, Esteve Gudayol-Ferré, Joan Guàrdia-Olmos

**Affiliations:** ^1^Facultat de Psicologia, Universitat de Barcelona, Barcelona, Spain; ^2^Institute of Neuroscience (UB), The UB Institute of Complex Systems (UBICS), Barcelona, Spain; ^3^Facultad de Psicología, Universidad Michoacana de San Nicolás de Hidalgo, Michoacan, Mexico

**Keywords:** reliable change index, depression, remission, longitudinal, clinical significant change

## Abstract

**Background:** Although major depressive disorder is usually treated with antidepressants, only 50–70% of the patients respond to this treatment. This study applied Jacobson and Truax’s (1991) methodology (reliable change index, RCI) to a sample of depressive patients being treated with one of two antidepressants to evaluate their functioning and the effect of certain variables such as severity and age.

**Method:** Seventy-three depressive patients medicated with Escitalopram (*n* = 37) or Duloxetine (*n* = 36) were assessed using the Hamilton depression rating scale over a 24-week period.

**Results:** They indicate that the RCI stabilizes in an absolute way starting in week 16, and it is not until week 24 that all of the patients become part of the functional population. We found limited statistical significance with respect to the RCI and the external variables.

**Conclusion:** Our study suggests the need to accompany the traditional statistical methodology with some other clinical estimation systems capable of going beyond a simple subtraction between pre and posttreatment values. Hence, it is concluded that RCI estimations could be stronger and more stable than the classical statistical techniques.

## Introduction

Major depressive disorder (MDD), which is a highly prevalent and disabling disorder that is associated with major personal and societal costs, is the second cause of disability worldwide. This worldwide prevalence and disability justifies the importance of investigating the treatments available (Driessen et al., 2015). While depression is more prevalent in women, single individuals, and those of low socioeconomic status (Schulz and Arora, 2015), MDD frequently co-occurs with other psychiatric disorders (Holma et al., 2014).

While there are several effective treatments for depression, antidepressant drugs are the most common. However, up to 60% of the patients with MDD do not respond satisfactorily to an initial antidepressant trial (Kasper and Montgomery, 2013), leaving them half symptomatic and functionally impaired. Unfortunately, the choice of antidepressant for a patient is a matter of trial and error, as there is little empirical evidence for choosing one treatment over another (Bruder et al., 2014).

The patients must remain on their antidepressant for up to 8 weeks before knowing whether they will respond to that particular drug. This risk may prolong distress, increase morbidity and mortality, and increase the burden on health professionals (Kemp et al., 2008). For this reason, several researchers have tried to find variables that could predict antidepressant responses and grouped them into subject, severity, and clinical variables (Gudayol-Ferré et al., 2010).

Regarding the subject variables, we examine the relationships between variables such as age, years of formal education, premorbid intelligence, and genetics and MDD-related cognitive impairment. For the effects of age on remission, Reynolds et al. (1998) examined treatment outcome differences when considering age at the onset of the first lifetime episode of recurrent major depression in elderly patients. They found that early-onset patients required 5–6 weeks more than late-onset patients before achieving remission, and they also exhibited a higher proportion of suicide attempts.

Furthermore, high levels of education correlate with low levels of depression. It has also been found that lower IQ scores are associated with increased risks of schizophrenia, severe depression, and other non-affective psychoses (Zammit et al., 2004).

Three types of genetic variables are usually analyzed in relation to MDD, the first being genetic polymorphism. There exists a functional polymorphism in the 5HTTLPR serotonin transporter gene that is associated with the polymorphic region. This gene produces two alleles, namely, a long (l) allele and a short (s) allele. The short allele (s) is associated with a higher risk of psychotic symptoms during a depressive episode (Stamm et al., 2013).

More recently, an A/G SNP (rs25531) was identified within the insertion fragment of the 5HTTLPR region that defines a triallelic system (La, Lg, and S), among which La is the highest expressing allele. Some studies have shown that this allele presents a better response to an SSRI (selective serotonin reuptake inhibitor) (Yu et al., 2002). Finally, we find the COMT gene (the catechol-*O*-methyltransferase), which has the functional Val108/158Met polymorphism that produces two alleles, namely, the Val (allele G) and the Met (allele A) variants. The Val gene has a small indirect effect on the clinical response to citalopram. Moreover, the Met/Met gene has a higher risk of non-remission in 6–8 weeks (Arias et al., 2006). Regarding disease severity, there are several factors, such as age at the first episode, number of past depression episodes, and number of comorbid anxiety disorders, that can influence the remission of depression.

As for the age at the first episode, Dew et al. (1997) reported that elderly patients who were older when they experienced their first depression episode exhibit a more rapid and sustained response profile. The recurrence of depression episodes is another important factor that may influence the remission of depression. Many individuals with recurrent MDD fail to show complete interepisodic recovery and, in fact, show more chronic episodes (Wilson et al., 2014). Beyond the high rates of coexistence, comorbid anxiety has often been cited as a clinically relevant problem owing to its impact on acute treatment response in late-life depression. Thus, several studies have found that a greater severity of anxiety symptoms is associated with an increased risk of withdrawal from treatment, a decreased response to acute antidepressant treatment, and longer response and remission times (Steffens and McQuoid, 2005).

Finally, with respect to the third group (clinical variables), the psychopharmacological treatment being administered and the speed of response exhibited by the subjects (fast, slow, or non-responders) are considered. It has been determined that escitalopram is better tolerated and superior to duloxetine in the acute treatment of MDD (Wade et al., 2007). With regard to the speed of response, there exist various patterns of recovery from the illness with an important variability among patients regarding both the speed of response and remission (Stassen et al., 2007). Usually, the patients are classified as fast, oscillating, or slow (Gudayol-Ferré et al., 2013).

To evaluate the efficacy of the treatments, statistical comparisons between the mean changes resulting from the treatments (Jacobson and Truax, 1991) are used. In neuropsychological and clinical assessments, in many situations, a person completes the same psychological measures on two or more occasions. In such a situation, test score gains are hypothesized to exist independent of actual changes in a person’s ability on the construct being measured by the test (Calamia et al., 2012; Marco et al., 2014; Wolpert et al., 2015; Egger et al., 2016a,b; Müller et al., 2016)

Nonetheless, the reliable change index (RCI) approach is still uncommon among clinical researchers not only in psychology, but in most health fields. This approach exhibits two properties that make it especially appealing. The first is the individualized study of each patient’s clinical progression, which entails not only the specific knowledge of their clinical condition but also implies changing from a contrast with respect to the mean (commonly criticized with no counterproposals) to a contrast with respect to the patients themselves. Second is the possibility to classify patients as functional or dysfunctional, which allows us to conduct a rare second analysis. It does not suffice to know whether the mean or median of a clinical indicator is significantly or marginally improved when compared to their pretreatment value. Rather, it must be known which variables intervene in the non-change evidence and which variables influence change. Put more succinctly, the RCI approach allows for the analysis of the implications of the complementary variables when identifying those patients who have improved and those who have not. It is not reasonable to think that the application of one treatment is the only source of variation, especially in applied clinical research where primary and secondary controls are limited.

Despite the current precedents in applying the RCI (Moleiro and Beutler, 2009; Lilja et al., 2016; Beiwinkel et al., 2017; Brooks et al., 2017; Foki et al., 2018), it has not been used extensively. That is, it has not been used to generate a secondary analysis to search for variables that may become improvement catalysts or hindering variables beyond the effect of the treatment.

The purpose of the current study is to identify which factors are related to remission in subjects diagnosed with MDD and undergoing psychopharmacological treatment, i.e., duloxetine or escitalopram. The level of remission is defined as the estimation of clinically significant change by Jacobson and Truax (1991), and hence, we identify the following specific objectives:

- To study the level of remission based on clinically significant changes in a 24-week follow-up.
- To analyze the effects of applying three cut-off points used in the estimations of the clinically significant change to determine which subjects become part of the functional population after the treatment and to identify the temporal moment in which this change occurs.
- To study the effects of age, the recurrence of the depression episodes, the level of education, the premorbid intellectual coefficient, the comorbid anxiety disorders, the genetic polymorphism, the age at the first episode and the response pattern of the drugs in the clinically significant change.
- To study whether the two types of drugs, i.e., duloxetine and escitalopram, are related, in any way, to the clinically significant change.

## Materials and Methods

### Design

The sample was comprised of 73 depressive patients who were recruited for the study. The treatments were assigned pseudorandomly, such that the first subject recruited in a subgroup was administered one treatment, while the second subject received the other treatment. One group received 60 mg per day of duloxetine (*n* = 37), and the other group received 10 mg per day of escitalopram (*n* = 36). Consequently, the final design was an experimental factorial mixed design with matched groups, one receiving the duloxetine treatment and the other receiving the escitalopram treatment.

### Participants

The present study is a 24-week follow-up of subjects who participated in a clinical trial designed to assess the possible effects of treatments using SSRIs and SNRIs (selective noradrenaline reuptake inhibitors) on cognitive functions in antidepressant drug-naïve patients with MDD (Herrera-Guzmán et al., 2009). The 101 patients were included in the study if they met the DSM-IV criteria for MDD. The inclusion criteria were as follows: (i) a diagnostic confirmation based on the mini-international neuropsychiatric interview (MINI) (Sheehan et al., 1998); (ii) a score of 18 points or higher on the Hamilton depression rating scale (HAM-D-17) (Hamilton, 1967); (iii) an age between 20 and 50 years; and (iv) a finding of being antidepressant-drug naïve and free of other psychopharmacological compounds. Patients were excluded if they failed to meet the DSM-IV criteria or had a past history of any of the following disorders: posttraumatic, obsessive-compulsive, schizophrenia, psychotic, delusional, bipolar, or substance abuse. Similarly, subjects with any present or past disease involving the central nervous system were also excluded. Additional exclusion criteria included diabetes; hypertension; cardiac, hepatic, pulmonary, and renal disease; and systemic infectious diseases. Women entering the trial could not be pregnant, and they had to be free of any oral contraceptives. Patients were also excluded if they showed an inefficient response to the pharmacological treatment after 4 weeks, i.e., the response to treatment on the treated (TOT) was defined as a 50% or greater reduction on the HAMD-17 in week four compared to the HAM-D-17 baseline score. Of the 101 recruited patients, 73 completed the study, while 28 abandoned or withdrew from the study for various reasons. Consequently, the escitalopram group was comprised of 37 patients, and the duloxetine group included 36 patients. All participants completed the registration forms during the follow-up weeks. The sample’s demographic information is presented in **Table 1**. The homoscedastic condition is met in all cases, and no differences between the variables are apparent. The protocol was approved by the ethics committee of the Michoacán Mental Health Center (*Centro Michoacano de Salud Mental*, in Spanish).

**Table 1:** *T*-Test comparison between treatment groups of descriptive and clinical variables.

Variable	Escitalopram mean *(SD) n* = 37	Duloxetine mean *(SD) n* = 36	*t*-Test	*df*	*p*
Age	32.91 (8.73)	33.21 (8.61)	0.299	71	0.881
Years of formal education	11.63 (3.71)	11.40 (3.93)	0.260	71	0.795
WAIS III vocabulary score	33.86 (7.61)	33.24 (6.71)	0.368	71	0.714
HAM-D-17 baseline score	25.30 (4.04)	25.1 (5.28)	0.130	71	0.897
HAM-D-17 after treatment score	0.58 (0.87)	0.78 (1.52)	0.685	71	0.493
Age at first depressive episodes	21.72 (8.65)	19.21 (8.84)	1.223	71	0.225
Number of past depressive episodes	3.81 (4.76)	3.59 (3.47)	0.217	71	0.820
Number of comorbid anxiety disorders	1.11 (0.85)	0.86 (0.88)	1.207	71	0.231


*df, degrees of freedom; *p*, significance (*p* two tailed).*

### Instruments

All sampled participants were assessed by means of several psychological tests that assessed specific psychometric characteristics. These included the following

- Depression severity: Hamilton Reduced Depression Scale (HAM-D-17) (Bagby et al., 2004).
- Premorbid intellectual verbal functioning: WAIS III vocabulary subtest (Wechsler, 1997).
- Demographic and clinical questionnaire: a questionnaire generated *ad hoc* for this procedure.
- Genotyping: Genomic DNA was extracted from venous blood samples using a standard protocol. The Val108/158Met (rs4680) COMT polymorphism was genotyped by 5′-exonucleaseassay using TaqMan SNP genotyping assay-on-demand. A 5HTTLPR triallelic system was also performed.

### Procedure

Once the recruited patients were assessed, the administration of the assessments occurred throughout the 24-week period to patients in the two groups, i.e., the 60 mg/day of duloxetine group (36 patients) and the 10 mg/day of escitalopram group (37 patients). All patients were clinically assessed by the psychiatrist in weeks 4, 8, 12, 16, 20, and 24 to control the clinical efficacy and safety of the treatment. At the end of the trial, all subjects were assessed again using the HAM-D-17 scale to evaluate the effects of SSRIs and SNRIs. The analysis of the clinical data was blind to the subjects’ genotype, and the genotype analysis was blind to the subjects’ clinical data.

In all cases, regular clinical supervision and reliability estimates were conducted between investigators of the administration and correction processes of the scales. In all cases, the reliability values exceeded.92.

### Statistical Analysis

Traditional methods used to evaluate treatment efficacy have been deemed problematic since the 1980s (Ronk et al., 2016). As a result, Jacobson and Truax (1991) proposed a RCI:

$$RCI=\frac{x_{2}-x_{1}}{S_{diff}}$$

where *X_2_* is the posttest score for each patient, *X_1_* is the pretest score for the same patient, and *S_diff_* describes the spread of the distribution of change scores that would be expected if no actual change occurred. An RCI over 1.96 would be unlikely to occur (*p* < 0.05) without actual change.

They proposed a methodology to classify the subjects into functional or dysfunctional populations according to three cut-off points following the empirical criteria presented in the next section. To analyze the effect of the complementary variables (age, premorbid intelligence, genotyping, type of remission, drug, age at first episode, number of past episodes, comorbid disorders, and years of formal education) on the observed distribution of the RCI, we used nonparametric (Mann-Whitney’s U or Kruskal-Wallis’ H) and parametric (Student’s *t*, Snedecor’s F, ANOVA, or Pearson’s r_xy_) estimates and contrasts, depending on the conditions of the application and the characteristics of the observed distributions.

## Results

To estimate the RCI we used Jacobson and Truax’s (1991) methods. However, being only interested in one tail of the normal distribution, we used the reference value to measure the clinically significant change of 1.64 as a unilateral contrast result. The following values were used: *S_diff_ = 1.737, r_xx_ = 0.81, and S_E_* = 1.228 i *S_1_* = 2.818. **Table 2** presents the descriptive values of the estimations of the RCI for every week.

**Table 2:** Descriptive statistics of the reliable clinical index for every week of treatment.

	*n*	Minimum	Maximum	$\bar{x}$	*SD*
RCIH2	68	-13.82	0.00	-5.14	2.61
RCIH4	63	-12.09	-1.15	-6.95	2.50
RCIH6	60	-13.24	0.00	-7.71	2.85
RCIH8	59	-14.39	-1.73	-8.70	2.67
RCIH10	14	-9.21	-2.30	-6.37	1.94
RCIH12	58	-13.82	-0.58	-9.30	2.80
RCIH16	51	-14.39	-3.45	-9.99	2.41
RCIH20	46	-15.54	-4.03	-10.69	2.43
RCIH24	44	-16.12	-5.76	-11.29	2.15


*$\bar{x}$, mean; SD, standard deviation; RCI, reliable change index.*

**Table 2** presents a clinically significant change in weeks 24, 20, 16, and 10. In the other weeks there is no evidence of such change for any of the participants. It is further noted that in week 12, one subject dropped with respect to week 10 and then improved in week 14. This improvement can be explained by the loss in week 10 (*n* = 14).

The mean and the standard deviation of the control population were estimated as a mean of the observed means reported in the different papers. This estimation was obtained from Herrera-Guzmán et al. (2010a,b) (*n* = 37), who obtained a mean of 3.2 and a standard deviation of 1.91; Gilley et al. (1995) (*n* = 54), who obtained a mean of 6.4 and a standard deviation of 6.7; and Reynolds and Kobak (1995) (*n* = 118), who obtained a mean of 3.67 and a standard deviation of 3.11.

The mean of our sample’s dysfunctional population is 21.51, and its standard deviation is 2.818. To calculate the Hamilton scale cut-off point for the distribution of the dysfunctional population, we used the distribution from the sample studied. Thus, the following values were obtained according to the previously discussed three criteria.

$$\begin{array}{l} \text{A }({\bar{x}}_{dysfunctional}-2\text{SD})=15.87,\\ \text{B }({\bar{x}}_{functional}+2\text{SD})=9.93,\\ \text{and C ((}{\bar{x}}_{functional}+{\bar{x}}_{dysfunctional}\text{)/2)=12.90} \end{array}$$

**Table 3** displays the frequency and percentage of switching to the functional population following the indications of the three cut-off points. Considering the type of disease being treated, the most proper cut-off point would be *c* because, with respect to this disorder, both populations overlap. As presented in **Table 3**, in week 24, all subjects were within the functional population.

**Table 3:** Frequency and percentage of switch to the functional population following the cut-off points A, B, and C.

		Cut-off point A frequency (%)	Cut-off point B frequency (%)	Cut-off point C frequency (%)
H2	Switch functional	50 (70.4)	17 (23.9)	28 (39.4)
	Non-functional	18 (25.4)	51 (71.8)	40 (56.3)
	Missing	3 (4.2)	3 (4.2)	3 (4.2)
H4	Switch functional	58 (81.7)	33 (46.5)	44 (62.0)
	Non-functional	5 (7.0)	30 (42.3)	19 (26.8)
	Missing	8 (11.3)	8 (11.3)	8 (11.3)
H6	Switch functional	55 (77.5)	41 (57.7)	50 (70.4)
	Non-functional	5 (7.0)	19 (26.8)	10 (14.1)
	Missing	11 (15.5)	11 (15.5)	11 (15.5)
H8	Switch functional	55 (77.5)	51 (71.8)	54 (76.1)
	Non-functional	4 (5.6)	8 (11.3)	5 (7.0)
	Missing	12 (16.9)	12 (16.9)	12 (16.9)
H10	Switch functional	11 (15.5)	5 (7.0)	8 (11.3)
	Non-functional	3 (4.2)	9 (12.7)	6 (8.5)
	Missing	57 (80.3)	57 (80.3)	57 (80.3)
H12	Switch functional	55 (77.5)	51 (71.8)	53 (74.6)
	Non-functional	3 (4.2)	7 (9.9)	5 (7.0)
	Missing	13 (18.3)	13 (18.3)	13 (18.3)
H16	Switch functional	51 (71.8)	47 (66.2)	50 (70.4)
	Non-functional		4 (5.6)	1 (1.4)
	Missing	20 (28.2)	20 (28.2)	20 (28.2)
H20	Switch functional	46 (64.8)	44 (62.0)	45 (63.4)
	Non-functional		2 (2.8)	1 (1.4)
	Missing	25 (35.2)	25 (35.2)	25 (35.2)
H24	Switch functional	44 (62.0)	44 (62.0)	44 (62.0)
	Missing	27 (38.0)	27 (38.0)	27 (38.0)


Switch functional: patients who, according to the cut-off points, switched to the functional population. Non-functional: patients who, according to the cut-off points, remained in the dysfunctional population. Missing: patients who were lost at a specific week.

The bivariate analysis was based on the RCI calculated above and the variables that could have modulated this remission, specifically, subject, severity, and clinical variables. However, we are only interested in the weeks with unremitting subjects, including week 12.

**Table 4** presents the bivariate analysis of the quantitative subject variables. In all cases, a Pearson correlation was conducted. As evidenced by the table, none of the analyses are statistically significant. Therefore, we can assume that none of these external variables affect the RCI estimation.

**Table 4:** Pearson correlation, degrees of freedom, *p*-value and effect size of the bivariate analysis between the RCI in weeks 12, 10, 8, 4, and 2 and the quantitative variables of the subject.

RCI	Age			Years of formal education			Premorbid intelligence		
	*r_xy_*	*df*	*p*	*r_xy_*	*df*	*p*	*r_xy_*	*df*	*p*
Week 12	-0.007	69	0.961	0.017	69	0.898	-0.004	69	0.975
Week 10	Insufficient sample size (*n* = 14)							
Week 8	0.054	69	0.687	-0.140	69	0.291	-0.120	69	0.365
Week 6	0.000	69	0.997	-0.056	69	0.673	-0.120	69	0.362
Week 4	0.088	69	0.492	-0.052	69	0.688	-0.116	69	0.367
Week 2	-0.105	69	0.395	0.023	69	0.852	-0.198	69	0.105


RCI, reliable change index; *r*_*xy*_, Pearson’s correlation; *df*, degrees of freedom; *p*, significance (*p* two tailed).

**Table 5** displays the bivariate analysis of the RCI in different weeks and the genetic polymorphism. **Table 6** presents the bivariate analysis of the RCI in the different weeks and the Rs25531. **Table 7** presents the analysis of the RCI with the COMT gene. As noted from the *p*-values, there is no significant relation in any week.

**Table 5:** Mean, SD, bivariate analysis (ANOVA/*Kruskall Wallis test*), and homogeneity test of the RCI in all the weeks and the variable genetic polymorphism.

RCI	Poly.	$\bar{x}$ *(SD)*	Contrast	*df*	*p*
Week 12 (*F* = 3.830, *p* < 0.05)^∗^	ss	-9.11 (3.69)	*H* = 0.340	2	0.843
	sl	-9.60 (1.82)			
	LL	-8.95 (2.49)			
Week 10	Insufficient sample size (*n* = 14)				
Week 8 (*F* = 3.536, *p* < 0.05)^∗^	ss	-8.66 (3.41)	*H* = 0.274	2	0.872
	sl	-8.91 (2.17)			
	LL	-8.25 (1.49)			
Week 6 (*F* = 1.869, *p* = 0.164)^∗^	ss	-7.76 (3.43)	*F* = 0.601	2/56	0.552
	sl	-7.99 (2.42)			
	LL	-6.78 (2.32)			
Week 4 (*F* = 0.058, *p* = 0.943)^∗^	ss	-7.67 (2.46)	*F* = 1.788	2/60	0.176
	sl	-6.39 (2.40)			
	LL	-6.70 (2.68)			
Week 2 (*F* = 1.046, *p* = 0.357)^∗^	ss	-5.75 (2.75)	*F* = 1.189	2/60	0.311
	sl	-4.75 (2.70)			
	LL	-4.76 (1.76)			


*$\bar{x}$, mean; SD, standard deviation; RCI, reliable change index; ()^∗^, homoscedasticity analysis; *F*, statistical value of F of Snedecor; *H*, Kruskal-Wallis test; *df*, degrees of freedom; *p*, significance (*p* two tailed), ss, sl, and LL, different genotypes of the genetic polymorphism.*

**Table 6:** Mean, SD, bivariate analysis (*t*-test/*U de Mann Whitney test*), and homogeneity test of the RCI in all the weeks and the variable Rs 25531.

RCI	Rs 25531	$\bar{x}$ *(SD)*	Contrast	*df*	*p*
Week 12 (*F* = 6.657, *p* = 0.013)^∗^	LA	-9.25 (1.94)	*U* = 370.500	1	0.435
	No LA	-9.35 (3.50)			
Week 10	Insufficient sample size (*n* = 14)				
Week 8 (*F* = 5.44, *p* = 0.023)^∗^	LA	-8.67 (1.93)	*U* = 416.500	1	0.778
	No LA	-8.73 (3.27)			
Week 6 (*F* = 1.228, *p* = 0.261)^∗^	LA	-7.40 (2.47)	*t* = 0.831	58	0.409
	No LA	-8.02 (3.20)			
Week 4 (*F* = 1.392, *p* = 0.243)^∗^	LA	-6.49 (2.65)	*t* = 1.495	61	0.140
	No LA	-7.43 (2.28)			
Week 2 (*F* = 0.102, *p* = 0.751)^∗^	LA	-4.67 (2.46)	*t* = 1.592	66	0.116
	No LA	-5.67 (2.70)			


*$\bar{x}$, mean; SD, standard deviation; RCI, reliable change index; ()^∗^, homoscedasticity analysis; U, statistical value of U de Mann Whitney; *t*, *T*-Test; *df*, degrees of freedom; *p*, significance (*p* two tailed); LA and no LA, different genotypes of the Rs 25531.*

**Table 7:** Mean, SD, bivariate analysis (ANOVA/*Kruskall Wallis test*), and homogeneity test of the RCI in all the weeks and the variable COMT.

RCI	COMT.	$\bar{x}$ *(SD)*	Contrast	*df*	*p*
Week 12 (*F* = 0.737, *p* = 0.484)^∗^	AG	-9.14 (2.86)	*F* = 0.403	2/52	0.670
	GG	-9.74 (2.85)			
	AA	-8.88 (1.62)			
Week 10	Insufficient sample size (*n* = 14)				
Week 8 (*F* = 1.189, *p* = 0.313)^∗^	AG	-8.94 (2.64)	*F* = 0.840	2/53	0.438
	GG	-8.81 (3.00)			
	AA	-7.49 (1.41)			
Week 6 (*F* = 0.793, *p* = 0.458)^∗^	AG	-7.80 (3.10)	*F* = 0.410	2/54	0.666
	GG	-8.03 (2.84)			
	AA	-6.91 (1.88)			
Week 4 (*F* = 0.588, *p* = 0.559)^∗^	AG	-7.10 (2.24)	*F* = 1.704	2/57	0.191
	GG	-7.26 (2.61)			
	AA	-5.34 (2.99)			
Week 2 (*F* = 3.473, *p* = 0.037)^∗^	AG	-5.11 (2.60)	*H* = 0.994	2	0.608
	GG	-5.50 (3.04)			
	AA	-4.28 (0.732)			


*$\bar{x}$, mean; SD, standard deviation; RCI, reliable change index; ()^∗^, homoscedasticity analysis; *F*, statistical value of F of Snedecor; *H*, Kruskal-Wallis test; *df*, degrees of freedom; *p*, significance (*p* two tailed); AG, GG, and AA, different values of the genotype of COMT.*

**Table 8** presents the bivariate analysis between the age at first episode, the number of past episodes, the number of comorbid anxiety disorders, and the RCI in weeks 12, 10, 8, 6, 4, and 2. We also see a statistically significant relationship (*p* = 0.049) in **Table 8** between the variable age at first episode and the RCI in week two. Accordingly, in week two, the age at first episode influenced the remission of depression. As evidenced in the correlation, there exists a positive relation between these two variables, such that the younger someone is when they suffer their first episode, the better the prognosis, i.e., the higher the RCI. With respect to the number of past depressive disorders and the number of comorbid anxiety disorders, there is no statistical relation between these variables and the RCI in any week. Therefore, we conclude that neither of these variables influenced the remission of depression in this sample when calculated using the RCI).

**Table 8:** Pearson’s correlation, degrees of freedom, *p*-value, and effect size of the different quantitative variables of severity.

RCI	Age at the first episode				Number of past depressive episodes			Number of comorbid anxiety disorders		
Correlation	*r_xy_*	*df*	*p*	*R^2^*	*r_xy_*	*df*	*p*	*r_xy_*	*df*	*p*
Week 12	0.024	69	0.861	-	-0.137	69	0.303	-0.024	69	0.855
Week 10	Insufficient sample size (*n* = 14)							
Week 8	0.064	69	0.632	-	-0.062	69	0.640	0.048	69	0.716
Week 6	0.020	69	0.881	-	-0.052	69	0.691	-0.013	69	0.992
Week 4	0.091	69	0.479	-	0.135	69	0.291	0.016	69	0.899
Week 2	0.239	69	0.049	0.057	0.066	69	0.590	-0.215	69	0.078


*$\bar{x}$, mean; SD, standard deviation; RCI, reliable change index; *r_*xy*_*, Pearson’s; *df*, degrees of freedom; *p*, significance (*p* two tailed)*.

Finally, with respect to the clinical variables, **Table 9** presents the bivariate analysis between the types of pharmacological treatment administered and the RCI in the different weeks. As evidenced by the table, there is a statistically significant relation between the type of treatment and the RCI in week two (*p* = 0.001). The table indicates that the patients undergoing the escitalopram treatment have lower RCI absolute values and, therefore, a worse prognosis in week two. This suggests that the patients treated with duloxetine experienced a quicker clinically significant change than those treated with escitalopram, whereas in week four, both antidepressants were equally effective in producing a clinically significant change.

**Table 9:** Mean, bivariate analysis, and homogeneity test between the variables type of treatment and RCI in all the weeks of treatment.

RCI	Drug	$\bar{x}$ *(SD)*	Contrast	*df*	*p*	*r*
Week 12 (*F* = 1.121, p = 0.294)^∗^	Duloxetine	-9.18 (3.29)	t = 0.281	55	0.780	-
	Escitalopram	-9.40 (2.56)				
Week 10	Insufficient sample size (*n* = 14)					
Week 8 (*F* = 0.497, p = 0.484)^∗^	Duloxetine	-9.29 (2.85)	t = 1.278	56	0.207	-
	Escitalopram	-9.36 (2.56)				
Week 6 (*F* = 0.266, p = 0.608)^∗^	Duloxetine	-7.61 (2.90)	t = 0.251	57	0.803	-
	Escitalopram	-7.81 (2.89)				
Week 4 (*F* = 1.392, p = 0.243)^∗^	Duloxetine	-6.49 (2.65)	t = 1.495	61	0.140	-
	Escitalopram	-7.43 (2.28)				
Week 2 (*F* = 3.45, p = 0.068)^∗^	Duloxetine	-6.62 (2.53)	t = 3.45	60	0.001	0.165
	Escitalopram	-4.42 (2.24)				


*$\bar{x}$, mean; SD, standard deviation; RCI, reliable change index; ()^∗^, homoscedasticity estimation; *df*, degrees of freedom; *p*, significance (*p* two tailed).*

**Table 10** presents the bivariate analysis of the RCI in the different weeks and the type of remission and the speed of remission. There is a statistically significant relation in weeks eight (*p* = 0.021), six (*p* = 0.009), four (*p* < 0.001), and two (*p* < 0.001). In week eight, it is evident that the patients with a fast remission time experienced a more clinically significant change (higher RCI) than did non-responsive patients and patients with slower remission time. Similarly, the same results are found in weeks six, four, and two.

**Table 10:** Mean, bivariate analysis (ANOVA), and homogeneity test of the variables RCI in the different weeks and the type of remission.

RCI	Type of remission	$\bar{x}$ *(SD)*	Contrast	*df*	*p*	*r*
Week 12 (*F* = 1.407, *p* = 0.254)^∗^	Fast	-10.00 (2.607)	*F* = 2.125	2/52	0.130	-
	Slow	-9.65 (2.03)				
	Non-response	-8.33 (2.91)				
Week 10	Insufficient sample size (*n* = 14)					
Week 8 (*F* = 0.683, *p* = 0.510)^∗^	Fast	-9.92 (2.84)	*F* = 4.185	2/53	0.021	0.136
	Slow	-8.74 (2.23)				
	Non-response	-7.45 (2.43)				
Week 6 (*F* = 0.58, *p* = 0.522)^∗^	Fast	-8.87 (2.41)	*F* = 5.153	2/54	0.009	0.160
	Slow	-8.31 (2.38)				
	Non-response	-6.23 (2.88)				
Week 4 (*F* = 0.340, *p* = 0.713)^∗^	Fast	-8.63 (1.77)	*F* = 43.372	2/57	<0.001	0.603
	Slow	-8.13 (1.55)				
	Non-response	-4.18 (1.55)				
Week 2 (*F* = 5.234, *p* = 0.008)^∗^	Fast	-7.92 (2.26)	*H* = 34,419	2	<0.001	0.763
	Slow	-4.27 (1.30)				
	Non-response	-3.51 (1.20)				


$\bar{x}$, mean; SD, standard deviation; RCI, reliable change index; ()^∗^, homoscedasticity estimation; *F*, ANOVA test; *H*, Kruskal-Wallis test; *df*, degrees of freedom; *p*, significance (*p* two tailed); *r*, effect size.

## Discussion

According with our results, it is important to emphasize that the estimations clearly delineate the sustained remissions of the patients treated with both drugs. Generally, applying the different criteria demonstrates an equally high stability of this result, meaning that, regardless of the criterion used, the effect described herein remains unmodified. These results are congruent with others (Jacobson and Truax, 1991) not only in the field of MDD (Ozen et al., 2016) but in other fields as well (Schütze et al., 2014; Montero et al., 2015; Ruiz-Sancho et al., 2015; Skewes et al., 2015; de Oliveira et al., 2017).

By studying the effect over the course of 24 weeks, we obtained evidence that the RCI settled or stabilized in an absolute way beginning in week 16 and the remission state remained unmodified until the study’s end. The clinically significant change occurred after week 16 of the antidepressant treatment. Similarly, the results revealed slight differences between the classification criteria, thus confirming Jacobson and Truax’s (1991) proposal. Furthermore, as previously discussed, only in week 24 can we assume that all patients are part of the functional population. However, this consideration is subject to considerable debate given the psychometric criteria of the classification linked to the clinical diagnosis of MDD.

Similarly, the statistical results regarding the behavior of the RCI estimations are presented according to the different external variables. In this case, the results revealed limited statistical significance, which leads us to conclude that the levels of remission estimated based on the RCI are not dependent on external factors. Particularly, it is important to highlight the lack of a relationship between the genetic markers analyzed and the estimations of the RCI, a finding that is inconsistent with the findings of other studies (Arias et al., 2006). Furthermore, it is also important to emphasize that no link was found between the number of comorbid anxiety disorders and the estimations of RCI, another finding that is inconsistent with those of previous research (Gudayol-Ferré et al., 2010). Despite these inconsistencies, it is noted that, in week two, there is a statistically significant effect between the estimations of the RCI and the age at first episode. Specifically, the patients whose first episode occurred at a younger age demonstrated a higher absolute RCI value. According to the data obtained, our findings are not consistent with the data presented by Reynolds et al. (1998) because, in our sample, the younger patients presented better prognoses. However, this disagreement should be interpreted with caution given that the effect size is low (*R^2^* = 0.057). This effect swings from week four onward, where the values of the RCI are equal for both age groups. Our results also differ from those of Dew et al. (1997), perhaps due to the use of the RCI instead of the traditional methods. A statistical effect was also found between the RCI estimation and the speed of the response to the drugs in weeks eight, six, four, and two.

In addition to the study of external variables, it would be interesting to predict remission in patients treated with one specific antidepressant. Using the RCI estimations, we can study which external variables affect remission and individualize the pharmacological treatment for every individual, thus reducing the recurrence and the residual symptomatology of MDD.

Finally, as a brief summary, it is noted that the effects due to the RCI are extremely fast. Hence, it is assumed that these pharmacological treatments significantly reduce the MDD symptomatology, and thus, both treatments appear to be highly effective. In relation to the analysis of speed of response, our findings suggest the need of reconsideration of the classification criteria in order to establish more accurately the slow, fast, and non-respondent patients.

One last aspect to consider is the clinical impact this type of analysis promotes in applied clinical research. In the most basic research, its contribution is probably more limited since it consists of highly controlled trials. However, in the case of applied clinical research, the question is quite different. In fact, the group analysis presents serious limitations in terms of prognosis and evaluation of each patient and is usually conceptualized as a comparison with the control group. Clinically, it is debatable whether global comparisons respond to the characteristics of a particular patient. In the formulated proposal, the control is the patient himself and a theoretical distribution of functionality that does not depend on any other data. In the clinical evaluation, the subject is only checked against himself. It can be argued that our data do not reveal relevant statistical significance with external factors that may mediate or moderate the clinical effect. This is true, in our case, but it only indicates the possibility of conducting this type of contrasts and subsequent analyses, which, in applied research, are usually only performed at the group level rather than the level of the individual patient. The non-significance does not imply that the technique has not the necessary flexibility to be useful in applied clinical research.

With respect to limitations, while the simple sizes used are obviously small, this is justified by the fact that this is an applied investigation and it analyzes only the subjects who completed the entire 24-week protocol for every drug. Conversely, as the assignment of treatment to the patient was not strictly randomized, it is a source of possible bias. Finally, the design proposed may be much more interesting if at least one group had been incorporated to assess the RCI derived from a dual intervention, e.g., drug plus psychotherapy. To conclude, while the presence of a control group may offer some data on natural changes after 24 weeks, this project is not assessing the treatment’s efficacy, and therefore, this is a minor issue.

The limitations notwithstanding, our study has certain relevant properties. It is one of the earliest reports to assess a new use of the RCI analysis and the interpretation of the data-base. Specifically, this study applies Jacobson and Truax’s (1991). clinically significant change and conducts individual analyses of the variabilities that arise when examining the subjects’ responses to the two pharmacological treatments. Similarly, a statistical study was conducted on how other variables, such as age or comorbid anxiety disorders, affect the RCI.

## Conclusion

Based on the aforementioned RCI results, the following conclusions are drawn:

- The clinical results of the two treatments are especially good, and the RCI rapidly reflects the effects of the treatment.
- It is possible to clarify those subjects who experienced a clinically significant change and those subjects who did not.
- This effect was not sustained in all subjects because of the variability over the different weeks.
- The potential benefits of exploring predictors for individual antidepressants are considerable because patients can then be matched to the medication to which they are most likely to respond.

However, in our sample, this variability cannot be explained by the effect of different measured external variables such as age, premorbid intelligence, or genetic or clinical variables. That notwithstanding, the use of the RCI has proven useful for assessing the possible effects of those or other clinically relevant variables.

## Author Contributions

CC-M statistically analyzed the databases and applied the Jacobson and Truax (1991) (RCI). After conducting the statistical analysis, the author prepared the results extraction and composed the document. MP-C, JG-O, and EG-F supervised the protocol used to obtain the databases for the current study. Finally, MP-C and JG-O revised the paper and coordinated the study.

## Conflict of Interest Statement

The authors declare that the research was conducted in the absence of any commercial or financial relationships that could be construed as a potential conflict of interest.
